# Exploratory Data Analysis of Adverse Birth Outcomes and Exposure to Oxides of Nitrogen Using Interactive Parallel Coordinates Plot Technique

**DOI:** 10.1038/s41598-020-64471-w

**Published:** 2020-04-30

**Authors:** Aweke A. Mitku, Temesgen Zewotir, Delia North, Rajen N. Naidoo

**Affiliations:** 1 School of Mathematics, Statistics and Computer Science, University of KwaZulu-Natal, Durban, South Africa; 2 Discipline of Occupational and Environmental Health, School of Nursing and Public Health, College of Health Sciences, University of KwaZulu-Natal, Durban, South Africa; 3 Department of Statistics, Bahir Dar University, Bahir Dar, Ethiopia

**Keywords:** Medical research, Mathematics and computing

## Abstract

We propose that a parallel coordinates plot can be used to study multidimensional data particularly to explore discovery of patterns across the variables. This can assist researchers from the health sciences to visualize their cohort data with interactive data analysis. The study used data from Mother and Child in the Environment birth cohort in Durban, South Africa for the period 2013 to 2017 retrospectively registered. In this paper, we demonstrate that the exploration of multidimensional data with parallel coordinates plot and use of brushing using different colours assists with the identification of relationships and patterns. Parallel coordinates plot visualization facilitates the researcher’s skills to find trends, identify outliers and perform quality checks in large multivariate data. We have identified trends in the data that provide directions for further research, and illustrated thereby the potential of parallel coordinates plot to explore patterns and relationships of prenatal oxides of nitrogen exposure with multidimensional birth outcomes. The study recognized the co-occurrence of adverse birth outcomes among infants and these infants had mothers with moderate to high level of NOx exposure during pregnancy. Brushing using different colours facilitated the detection of patterns of relationships to perform basic and advanced statistical model-based analysis.

## Introduction

Data exploration techniques are important approaches for displaying multidimensional and multivariate datasets^1^, and to demonstrate the different ways that complex interactions among variables can be identified in images^2,3^. It is a method for identifying and confirming previously unknown and important patterns within data sets and an important philosophical approach to understanding one’s data^4^. Visual analytics is well developed for up to three variables (three-dimensional plots (3D plots)). Visual analytics for more than four covariates is complex and not well developed^5^. One of the most challenging stages in multivariate data analysis is to identify patterns and associations between a set of interrelated variables. Most typical data exploratory techniques fail to visualize multidimensional data^6^.

The typical exploratory data analysis techniques, such as bar charts, line graphs, histograms and scatter plots suit one or two-dimensional data^7^. However, visualization of complex multidimensional data is challenging for researchers. Box plots, scatter plots, heat maps, complex radial tree layout diagrams are among the most commonly used techniques to better explore multidimensional data^8^. The box-and-whisker plot is also useful in bivariate analysis, as it allows comparisons between different groups that results from categorical grouping variables^9^. Previous data exploration techniques relied mostly on scatter plot matrices^10^. However, it is not generalizable beyond three dimensions and making multivariate associations requires a large amount of screen space. Due to this, the problem of representing multidimensional data is a difficult and largely unsolved one.

**Figure 1:**
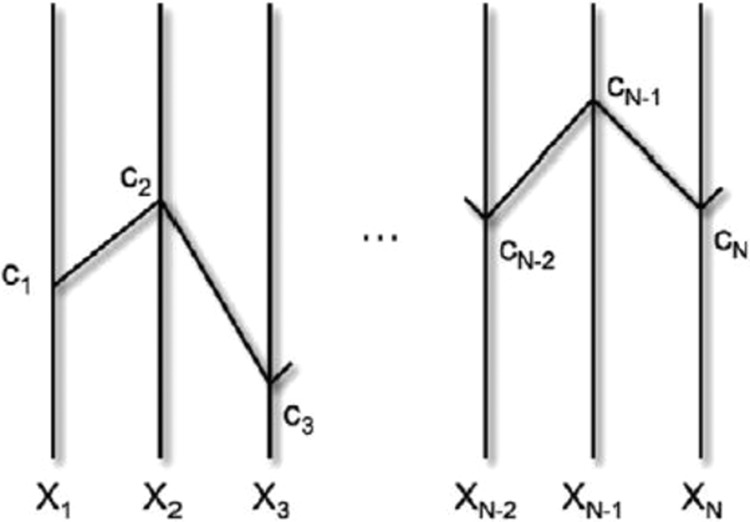
The polyline in parallel coordinates plot of the N-dimensional data with coordinates (*c*_1_, *c*_2_,…,*c*_*N*_)^13^.

The parallel coordinates plot (PCP) is one of the most recently applied visualizations of multidimensional datasets via a 2D mapping, wherein each dimension is drawn as *N* parallel axes next to each other and each data item is drawn as a polyline that connects its data values on parallel vertical axes^11^. The N axes in the parallel coordinates plot all have the same positive orientation as the Y-axis. In general, the technique produces a compact two-dimensional representation of *N*-dimensional data tuple *C* with coordinates (*c*_1_,*c*_2_,…,*c*_*N*_) by points (Fig. 1)^12,13^. This is an efficient way to place a large number of axes and to visualize the multivariate relations. PCP has become increasingly popular for interactive visualizations due to its ability to represent multidimensional data^14^. It is a powerful visualization technique used to visualize high-dimensional data in statistical sciences and data^11,15^ mining. The application of PCPs to health science problems, in particular, has been quite limited while it has been widely used in other science and engineering contexts^16,17^. Researchers have been working on improving this technique for better data investigation and user-friendly interaction by adding data clustering^18^, brushing^1,19^, etc. With these improvements, PCP becomes a very efficient technique for visualization relationships between selected neighbouring dimensions.

Visual data analytics with PCP is particularly advantageous in studying parameters across patients when clear hypotheses are unavailable in health science research settings^20^ and ultimately to improve the quality of healthcare^21^. For instance, PCP has been used to visualize complex and dynamic biochemical networks to better understand disease mechanism^22^ and for hypothesis generation in tumour tissue characterization^23^. Dai *et al*.^24^ also used PCP to explore risk factors by incorporating choropleth maps of mortality rates in a region. Due to the representation of each data point by a polyline, overplotting can have severe readability difficulties. Consequently, we are interested in extending the use of PCP as a tool that can be used in health sciences research to provide exploration for multidimensional health research data with easy-to-use graphical user interfaces such as brushing using different colours.

Previous scientific studies that aimed to investigate the relationship between air pollution and health outcomes have reached inconsistent findings in effect size or size of variability^25,26^. The study is located in the city of Durban, South Africa. The south of the city has well documented industrial emissions as it is highly industrialized, with two oil refineries, a paper mill, a sewage treatment plant, a major highway, landfill sites and many processing and manufacturing industries, in contrast to north Durban. Traffic-related air pollution studies generally have used nitrogen oxide (NOx) as an indicator, and land use regression models have frequently employed to model the fine scale spatial variability in NOx exposure^27^. The main aim of this study is to extend the use of PCPs with brushing using different colours to explore patterns and trends of annual prenatal oxides of nitrogen (NOx) exposure with multidimensional birth outcomes.

## Methods

### Data

This study used data from Mother and Child in the Environment (MACE) birth cohort, for the period 2013 to 2017. The main aim of the cohort is to examine the effects of environmental influences on the health of children born from a cohort of 1200 women attending ante-natal clinics. The children will be followed up to six years of age to evaluate respiratory health. The study enrolled a cohort of 996 women up to May 2017, from public sector antenatal clinics in the industry dense residential areas of south Durban and the residential areas of similar socio-economic status, but without industrial activity in north Durban. Of those enrolled for this study, 687 were followed up to infant delivery with complete data on the five dimensions of birth outcomes, which are reported in this paper. The postnatal data set of the cohort including multidimensional birth outcomes (gestational age, birth weight, birth length, head circumference and 5 minute APGAR score) was used. These indicators of health outcomes are often seen as important indicators of maturity and to an extent physical development of a fetus or newborn infant. The APGAR Score measures heart rate, muscle tone, and other signs to see if extra medical care or emergency care is needed. The test is usually given twice: once at one minute after birth, and again at five minutes after birth. In this study, we used the five minute APGAR score. In this study prenatal NOx exposure is predicted using a combined land use regression model for both south and north Durban and then the developed land use regression model was used to predict exposure at the residential addresses of a sample of study participants from the MACE cohort. This is described elsewhere^28,29^. The Mother and Child in the Environment Study was conducted under relevant guidelines, regulations, and research agreements. Ethical approval was obtained from the University of KwaZulu-Natal’s Biomedical Research Ethics Committee, and each participant provided informed consent, participation was voluntary and withdrawal from the study at any point was allowed.

### Parallel Coordinates Plot Development

We imported the nine data sets and the air pollution (NOx) data into the Statistical Analysis System (SAS) software, version 9.4. The data sets were merged in SAS, using the mother’s study identification number as a key variable. The data was edited and coded and then exported to SAS JMP software version 13.0 and R version 3.4.2 to produce the PCPs for data exploration. PCPs are techniques for visualizing the relationships between variables in multidimensional data sets. In these plots, vertical axes for each variable scaled to a common height are placed next to each other and connected with lines^30^. The patterns revealed by a plot strongly depend on the arbitrary placement of the corresponding axes. Accordingly, interactive axis rearrangement is essential to grasp all pairwise relations between the dimensions. Brushing is another vital interaction technique to select and advance a subset of multidimensional items based on the focus on top of the original view^31^.

In this study we have enhanced the PCP by tools such as axis reordering, brushing using different colours to address the issue of cluttering, disordered or crowded collection of graphical entities in PCP on a total of 687 pregnant women who were repeatedly followed-up for the MACE birth cohort. We have used reordering of dimensions of birth outcomes as a reduction of clutter and overlaps. Subset highlighting was implemented as a brushing interaction where selection only operates on aggregates. It was performed by dragging on the parallel coordinate plot. Typically, the selection of the lines that cross the rectangular area was specified by mouse dragging based on selection criteria. When the selected subsets are propagated to a PCP display, the lines of selected observations are coloured according to the classes the observations belong to, otherwise, the lines are shown in purple.

SAS JMP 13.0 supports a multi-step procedure of brushing, with an option to combine results of the last interaction with preceding selection by applying different logical operations (and, or, xor etc.). Then, the subsets of the data were selected interactively using different colours for comparing characteristics of subsets with different colours. The cut-off points for the classification of birth outcomes were based on the World Health Organisation (WHO) standards. The cut-off points used for brushing of low gestational age was at less than 37 completed weeks and high gestational age at 42 weeks and above, while 2500 g and 4000 g were used as cut- points for low and high birthweight respectively. Besides, the third and 97^th^ centiles of birth length and head circumference were used as a low and high cut-off points. For 5-minute APGAR score those below seven were selected to show lower APGAR status.

## Results

The study population covered a wide range of maternal ages, educational level, and further included families with different income levels. More than half of the infants born were males (52.3%) and most (64.0%) of the babies were vaginally delivered. Most mothers were single (80.8%), completed secondary schooling (55.3%) and had no personal income (45.3%). About 31.5% of the pregnant women in the cohort were found to be HIV positive with the higher prevalence in north Durban (42.4% vs 25.9%). The majority of the women were young with an average age of 26.03 years. The mean gestational age at birth in the cohort was about 39 weeks (SD: 2 weeks) while the mean birth weight, mean birth length and mean birth head circumference was 3107 g, 49.5 cm and 35.1 cm respectively. The median annual prenatal NOx exposure was about 34.23 µg/m^3^. The median annual prenatal NOx exposure for women from south Durban was about 36.97 µg/m^3^, higher than that of north Durban (26.35 µg/m^3^).

### Patterns and Relationships of Prenatal Air Pollution (NOx) Exposure Level on Adverse Birth Outcomes

**Figure 2:**
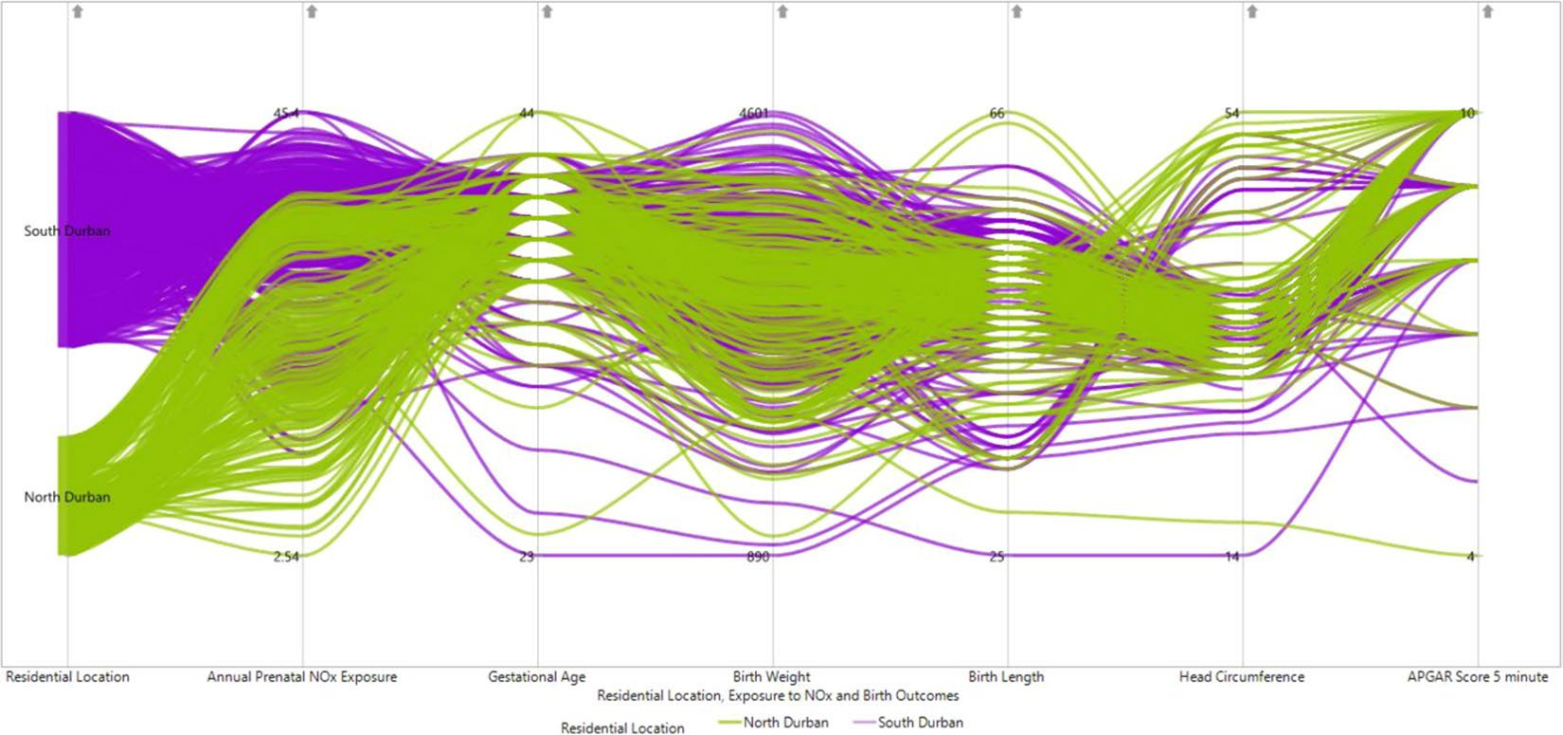
PCP of birth outcomes with prenatal NOx exposure and residential location (colour coded green for North Durban and blue for South Durban).

The PCP showed several patterns and relationships among adverse birth outcomes (preterm birth, postdates, low birth weight, overweight, low birth length, low head circumference and low 5 minute APGAR score). The exploration revealed that women from south Durban generally had a higher level of NOx exposure during pregnancy as compared to those from north Durban. The PCP did not show a clear pattern across all birth outcomes with regard to the level of prenatal NOx exposure, except for few outlying clusters of infants with higher sized head circumference and APGAR from north Durban (coded with green colour) (Fig. 2).

**Figure 3:**
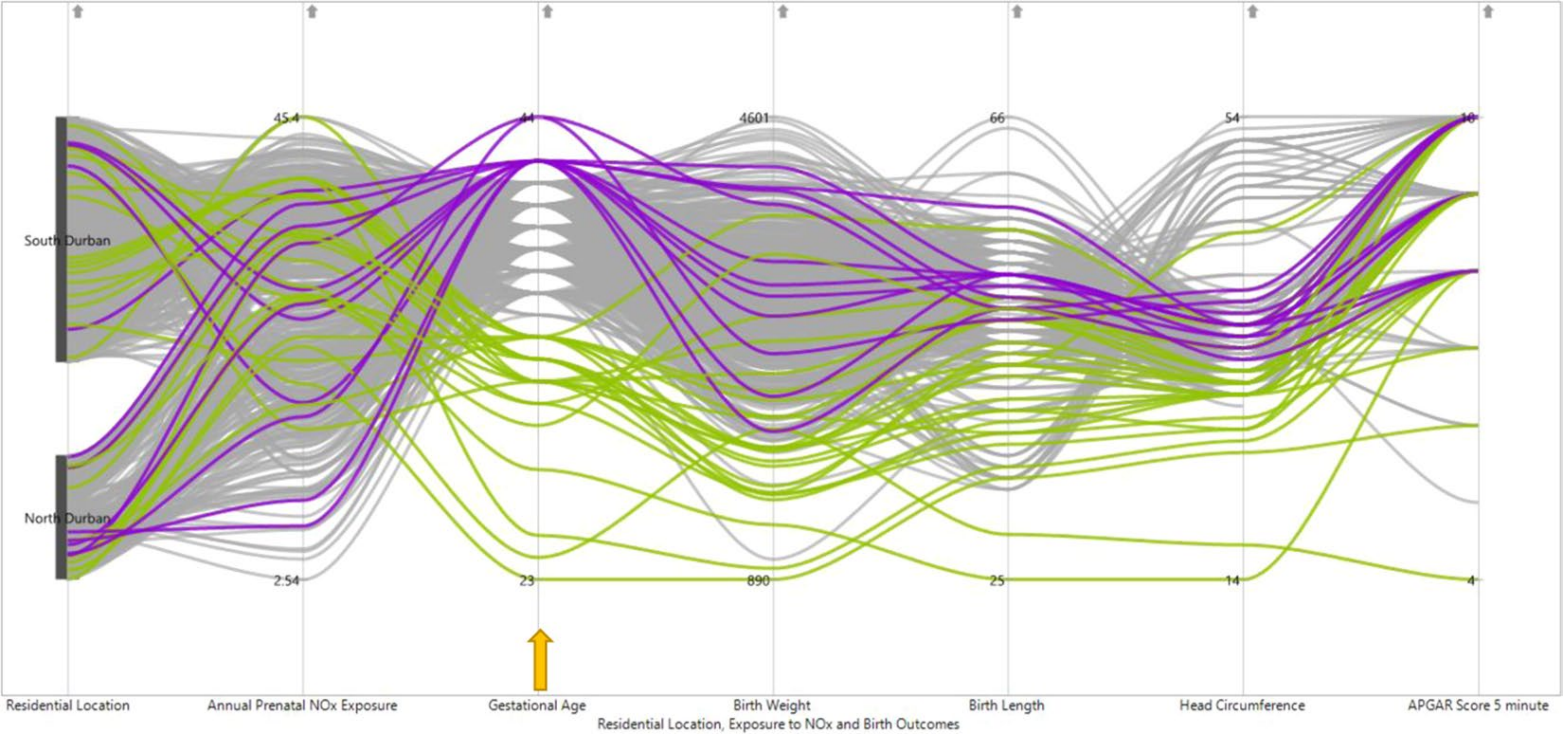
PCP for exploring pattern of relationships of prenatal NOx exposure levels with extreme gestational age (colour coded brushing of gestational age: green for preterm birth (below 37 completed weeks) and blue for postdates (42 weeks and above) indicated by the arrow).

**Figure 4:**
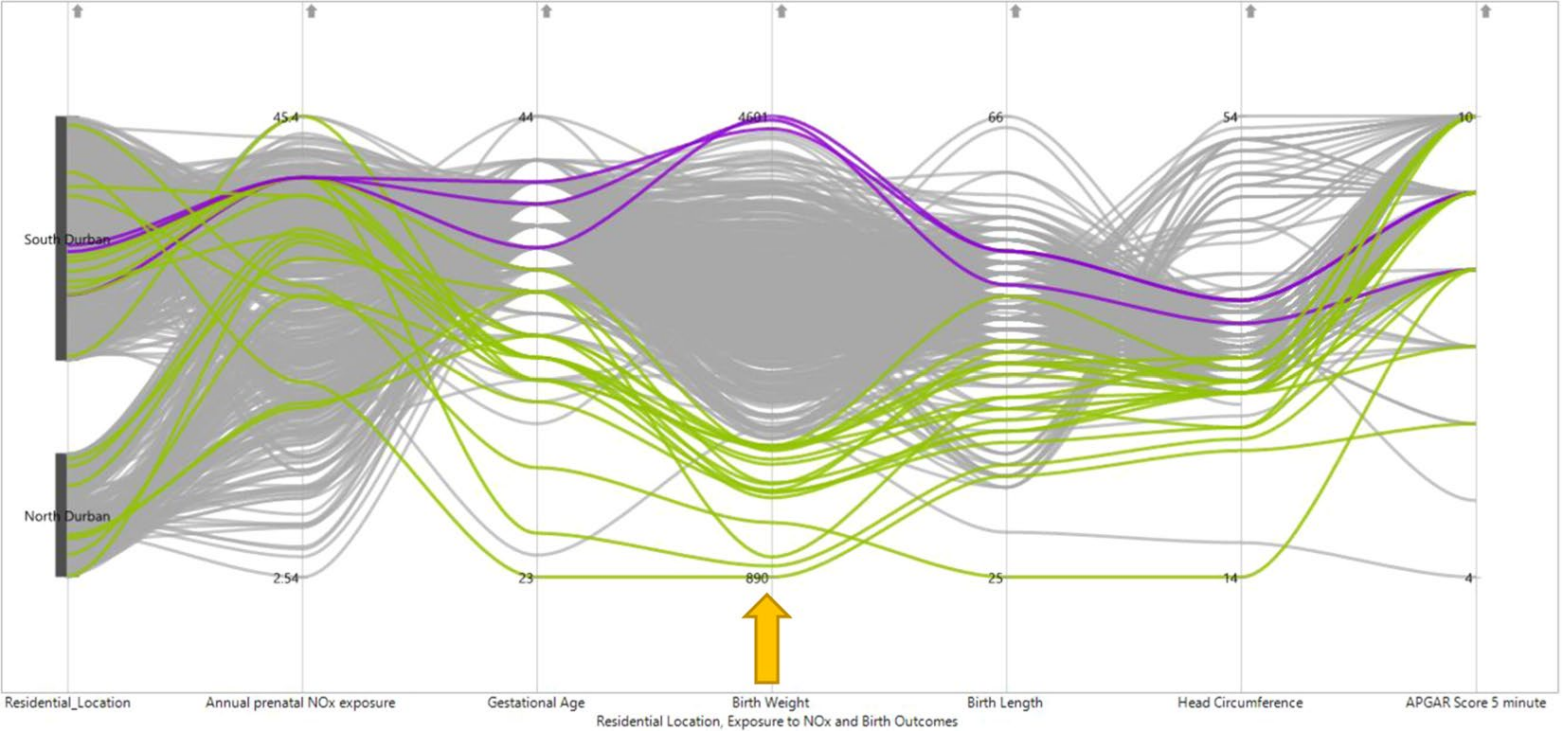
PCP for exploring the pattern of relationships of prenatal NOx exposure levels with extreme child birthweight (Colour coded brushing of adverse birth outcomes green for low birth weight (less than 2500 g) and blue for overweight (above 4000 g) indicated by the arrow).

**Figure 5:**
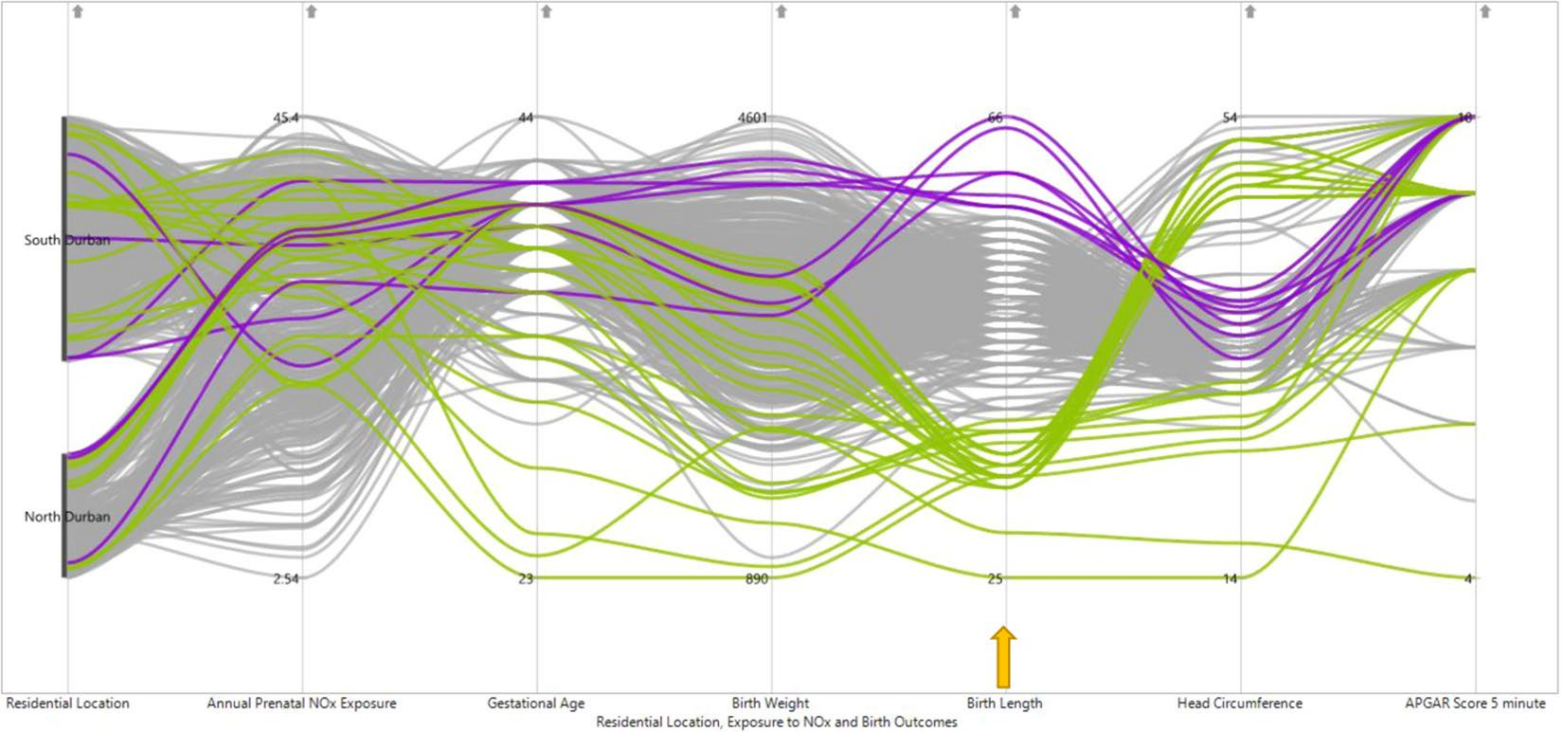
PCP for exploring pattern of relationships of prenatal NOx exposure levels with extreme child birth lengths (Colour coded brushing of adverse birth outcomes: green for low birth length (below the third percentile) and blue for high birth length (above 97 percentile) indicated by the arrow).

**Figure 6:**
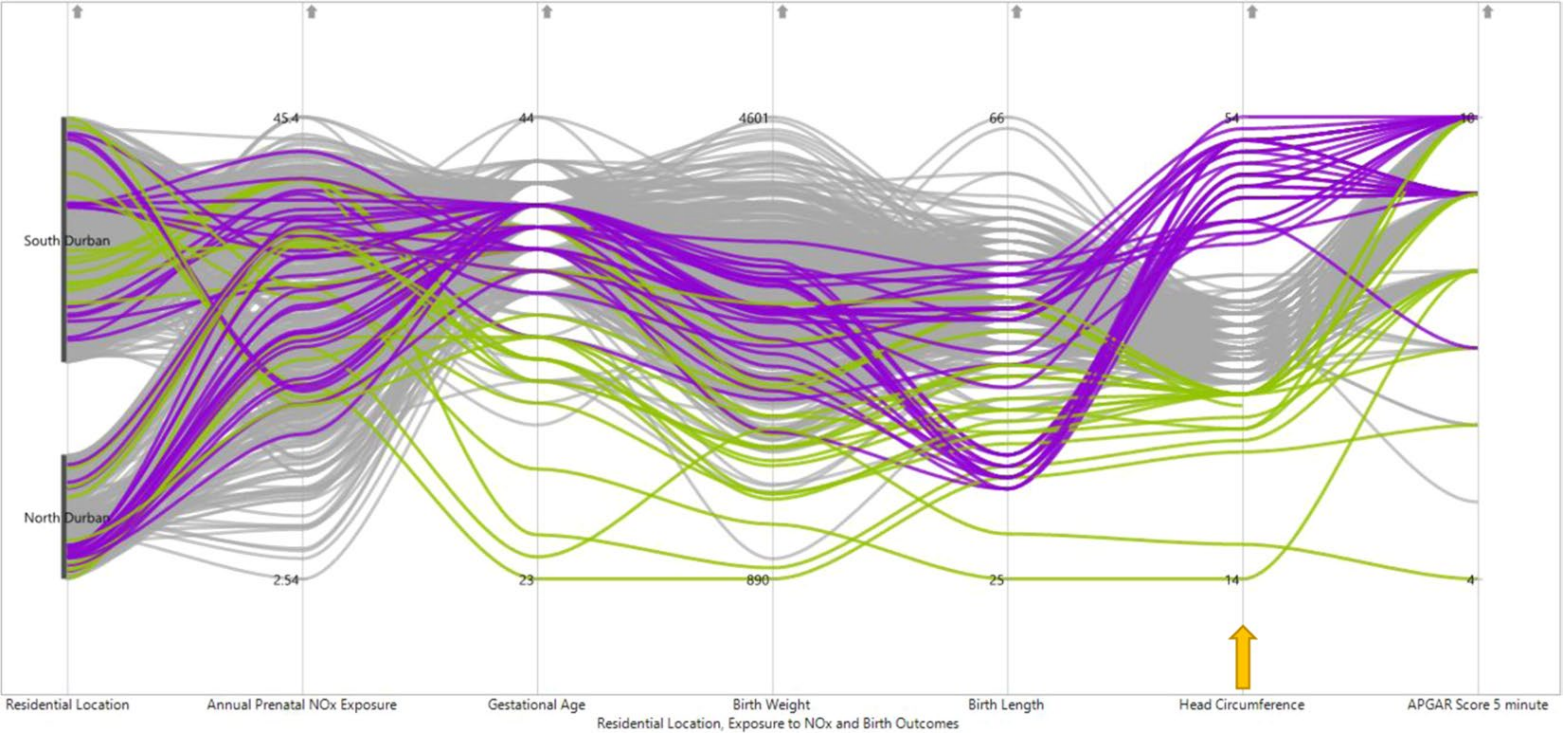
PCP for visualizing pattern of relationships of prenatal NOx exposure levels with extreme head circumference (Colour coded brushing of adverse birth outcomes green for low head circumference (below the third percentile) and blue for high head circumference (below 97 percentile) indicated by the arrow).

The PCPs in Figs. 3–6 have been configured through brushing and interactive techniques by colour coding, with green for lower and blue for higher values of birth outcomes. The graphs have six vertical axes, arranged from left to right along the X-axis, which represent the five dimensions of birth outcomes, level of prenatal NOx exposure and residential location. The units of measurement differ among these variables, including weeks, grams, centimetres and counts. Women with low gestational age at birth, tend to have relatively moderate prenatal NOx exposure levels (Fig. 3). However, higher gestational age of birth was observed with a high spread in the level of maternal NOx exposure during pregnancy. The majority of the women with preterm births, had a higher tendency of having an infant with low birth weight, birth length and head circumference, while women with postdated births seemed to have an infant with normal birth weight, birth length, head circumference and 5 minute APGAR score (Fig. 3).

Low birth weight was observed among women who experienced relatively moderate prenatal NOx values (Fig. 4). Similarly, the infants with low birth weight were seen to have a tendency of being preterm, with associated low birth length and smaller head circumference. Selection for low and high birth length (Fig. 5) demonstrated a trend of abnormal birth length with an average level of prenatal NOx exposure. The PCP further showed that low birth length seems to be observed with lower to normal gestational age and birth weight. On the other hand, low birth weight had shown a similar trend to both low and high head circumference (Fig. 5). A high variation in 5 minute APGAR score is observed for infants with low birth length.

**Figure 7:**
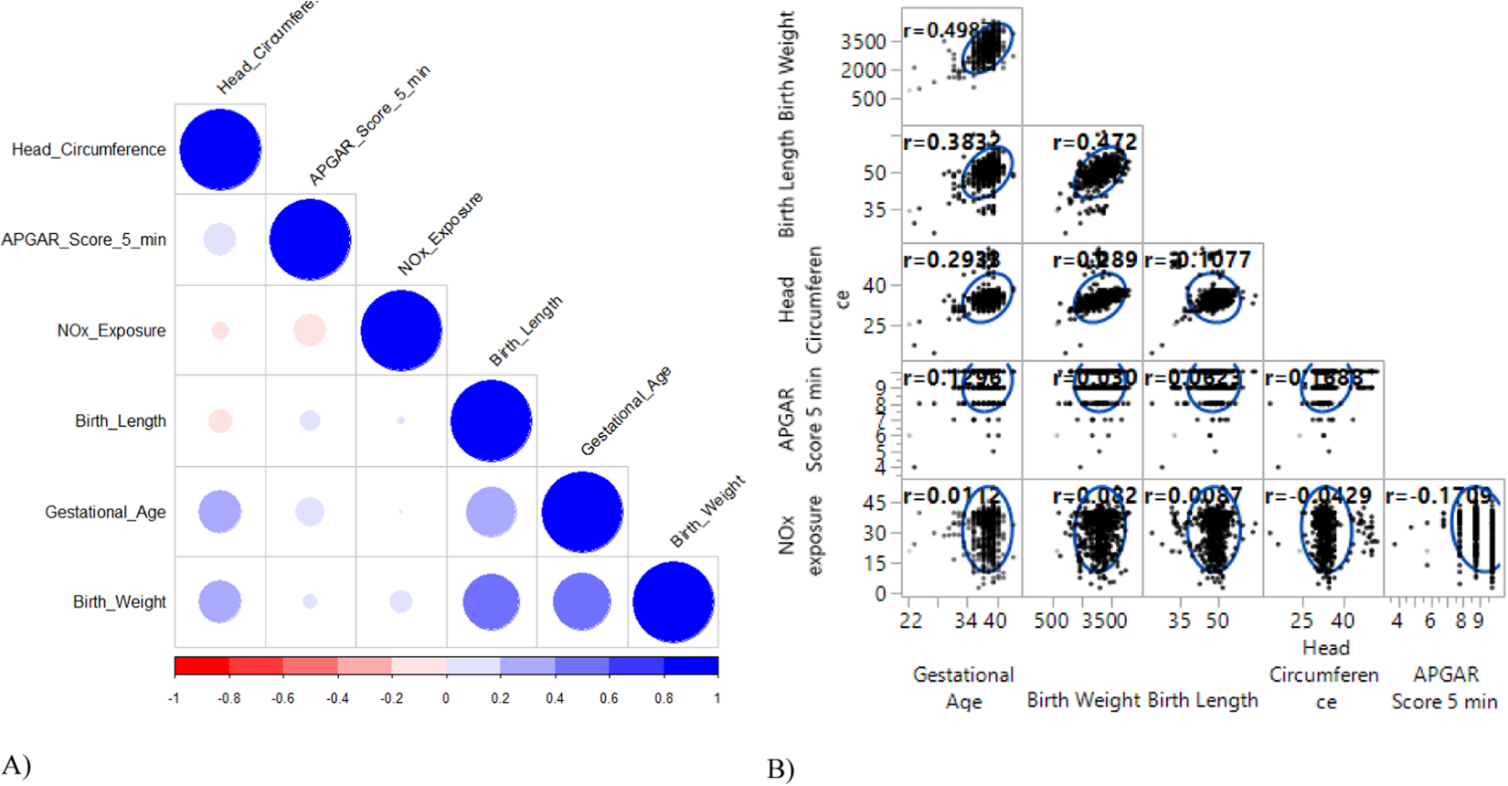
A. Correlation heat map and B. scatter plot matrix of dimensions of birth outcomes (Blue circles on the diagonal of the heat map (A) indicate correlations of the variables with themselves. Colour intensity and the size of the circle are proportional.

Women in the study who had babies with low or high head circumference infants at birth, tend to have had moderate prenatal NOx exposure levels (Fig. 6). From the PCP, it can be observed that infants with low head circumference had shown a higher tendency of being preterm, with associated low birth weight and low birth length. On the other hand, infants in the study with higher head circumferences tended to be normal in other birth outcomes, except for the fact that some had low birth lengths (Fig. 6). The correlation heat map (Fig. 7A) and the scatter plot matrix (Fig. 7B) have been further configured to display the correlations among birth outcomes. The scatter plot matrix reveals a positive relationship among gestational age, birthweight and birth length of a child and further indicates that these dimensions are weakly correlated with the 5 minute APGAR score. Head circumference correlated positively with gestational age and birthweight, but this relationship was the inverse for birth length and the APGAR score (Fig. 7B). The correlation heat map (Fig. 7A) confirmed a relatively weak, to moderately positive correlation among gestational age, birthweight, birth length and head circumference, except for the weak correlation between birth length and head circumference. This is similarly indicated in the scatter plot matrix (Fig. 7B).

## Discussion

Data exploration with PCP is advantageous in order to quickly examine patterns, trends, relationships and outlier detection in the multivariate data over traditional methods. It facilitates the 2D rendering of very complex datasets in a single image. PCP is a standard tool in exploratory data analysis that provides an overview of the relationships between the dimensions^5^. Interactive parallel coordinates complement the statistical tests, highlighting the relevant associations.

**Figure 8:**
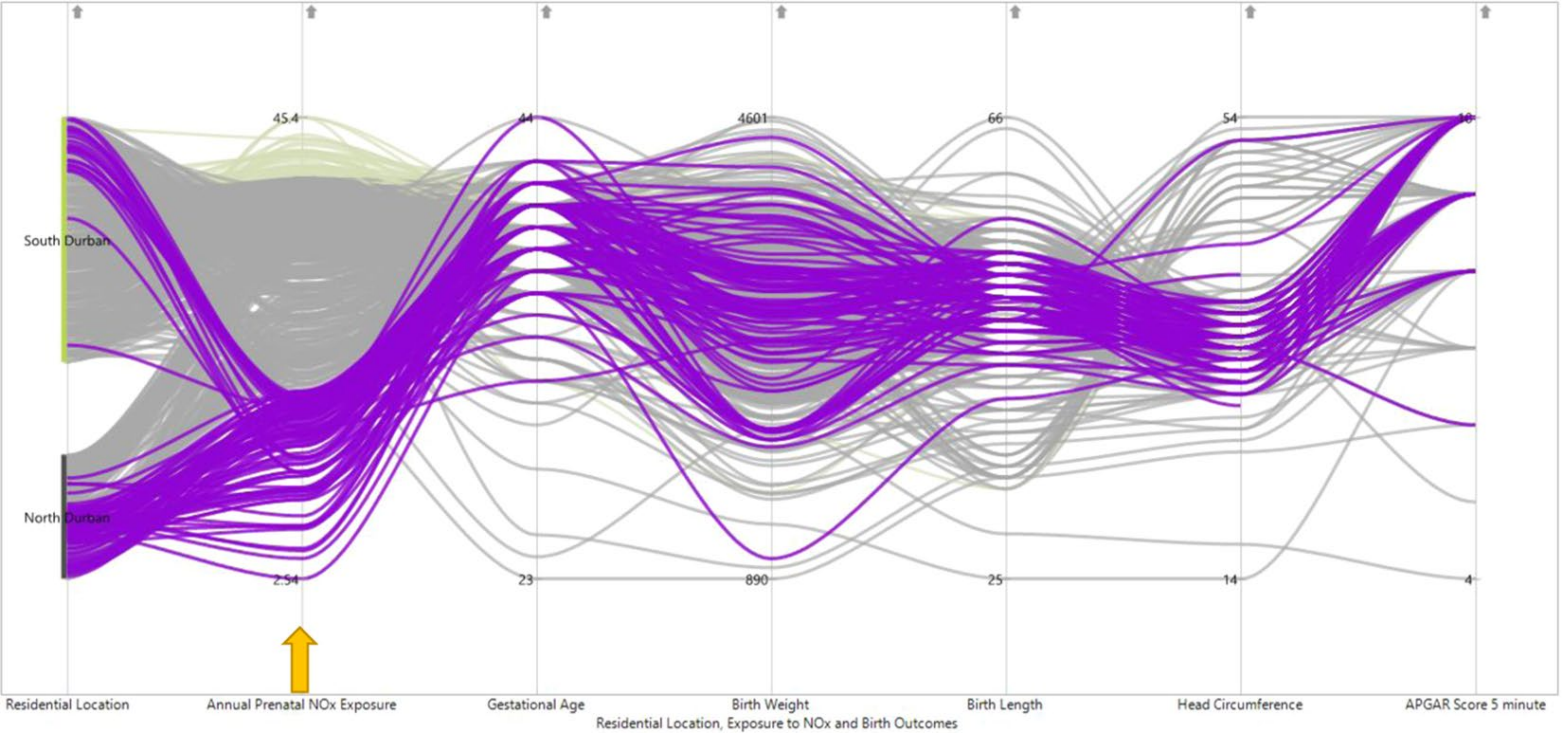
PCP for visualizing pattern of relationships of low (less than 20 µg/m^3^) NOx exposure levels with high APGAR scores (8, 9, 10) (Colour coded brushing of blue for low NOx exposure levels indicated by the arrow).

PCP is a suitable data exploration technique to make the data observations visible, as well as providing a technique to express the correlation between two characteristics of neighbouring axes^15^. The contributions of data exploration with PCP are twofold. Firstly, the exploratory visual techniques presented in this paper, the PCP, offers a simple method to use exploratory tools to investigate data relationships. For instance, we can follow the blue clusters displayed in the PCP of Fig. 8 and observe it is characterized by high APGAR scores for low exposure level of NOx (lower than 20 µg/m^3^). This indicates high APGAR scores of the infant was observed at a low level of pollution. Secondly, this technique is a more flexible data visualization tool than those identified in previous studies, where generally the exploration is limited to two, or at most three, dimensions^32,33^. In our study, we have used PCP on the five dimensions of birth outcomes and this provides us to see the trends between them and with NOx exposure levels. Many researchers showed that it is crucial to apply suitable interactive techniques with the dimensions of the data, to get insights about the multivariate^6,15,34,35^ data. In the case of this study, when we observe the blue coloured brushing of low head circumference in the PCP shown in Fig. 6, we can see that these infants tend to be preterm, low birthweight and low birth length. This provides an insight that adverse birth outcomes cannot be assumed independent rather there is a clear correlation among them. This implies that statistical models that consider associations are suitable instead of univariate models for analyzing these dimensions. The PCP visual analysis can accordingly be used by researchers to explore complex relationships in high-dimensional data before undertaking model building.

A common challenge is incorporating these exploratory data analyses within the platform of health sciences research without the need for complicated programming knowledge. Currently, there are high-level visual analysis tools that allow users to customize their visualizations. However, writing programming code in R demands high-level skills which are beyond most end users. The exploratory techniques of interactive PCP using SAS JMP employed in our study did not require programming skills and would expose the clinical research team to a common theme of identifying patterns and trends of data in contemporary bioinformatics^36^.

Data visualization with PCP is more effective for interactive exploration than traditional methods^37^. In this regard, we have followed the observations with adverse birth outcomes across all parallel axes of PCP based on the WHO cut-off points and trace the trends and relationships among themselves and with NOx exposure levels. However, it would be difficult to identify the trend of individual data value as well as the clusters of values of interest, for a large number of observations due to cluttering of data points. Our use of interactive brushing with different colours helped to easily identify the presence of correlation among different dimensions of birth outcomes. A study by Weaver also showed that PCP enables flexible exploration of various nuanced hypotheses through brushing using different colours, which is an interactive data selection^38^. Enhanced visualization of multidimensional data with multiple coordinated views, allow users to understand the same data set from different perspectives at once.

In this work, we illustrated the use of a parallel coordinates plot representation was advantageous compared to methods of graphical data analytics, such as the scatter plot matrix and the correlation heat map to explore useful relationships amongst multidimensional (5 dimensions) data on birth outcomes and levels of prenatal NOx exposure. Researchers used angular brushing to pick out data subsets with specific trends^39^ and directly manipulated parallel coordinates interactively to visualize the correlation between polyline subsets^40^. The brushing and interactive techniques, used in this study, are very effective tools in exploring the structures within the clusters particularly in identifying the consistent occurrence of adverse birth outcomes and their relationships with level of exposure to NOx. Many application domains have demonstrated where parallel coordinates and scatter plot matrix can be used for exploring the multivariate data^11,41,42^. For larger datasets, some user interactions are also incorporated in these techniques, such as selecting and filtering^43^.

A previous study by Inselberg^12^ discussed that parallel coordinates transform the search for relations among different attributes into a 2-D pattern recognition problem. In this study, we applied effective user interactions, which support and expand this knowledge discovery process. This exploration, along with associated user interactions, allows the identification of important correlation structures, demonstrates the pattern of relationships, provides an important framework for the visualization of relationships among the birth outcomes and further highlights some relationships with birth outcomes and level of prenatal NOx exposure. The correlation heat map and the scatter plot matrix identify correlations among dimensions of birth outcomes and exposure to NOx, revealed weak to moderate correlations among the four dimensions of birth outcomes, except for APGAR score, which was found to be weakly correlated.

In this work, the multidimensional graphical techniques PCP, with correlation heat map and scatter plot matrix, explored the patterns and relationships of the level of prenatal NOx exposure, with different profiles of birth outcomes. During the course of this exploration, it was noted that the potentially meaningful relationships were highlighted visually. This facilitates the assessment of multidimensional data simultaneously, in a single display, to ascertain the amount of variability that can be attributed to other specific variables and further has enables us to recognize the patterns and relationships between variables, which is beneficial to our model building due to the power of combining various data analytics techniques^44^.

As a limitation like many visualization techniques, readability, as well as efficiency of PCP, suffer when large datasets are displayed. For instance, with increasing samples, the PCP will become more cluttered and rendering these direct visualizations of the dataset is cumbersome and ultimately can be very difficult to interpret. To improve the exploration power of PCP, we further enhanced the visualization by varying colour and opacity, according to the local line density of the curves. For large sample size, to address cluttering issue, drawing curved edges instead of polylines^35^ and edge bundling^45,46^ are potential techniques. Our sample size is relatively modest, thus user interaction in the form of selection with brushing using different colours and axis reordering should furthermore be added to future studies that make use of large data sets and a large number of covariates. Axis reordering may be more challenging with an increasing number of covariates^47^. Previous researches^48,49^ showed dimension reduction as an alternative technique for multidimensional data visualization.

## Conclusion

We used interaction tools, such as axis reordering and brushing using different colours. This facilitates the detection of patterns of relationships in order to be more fully investigative in high volume, multidimensional data. We believe multidimensional data exploration has helped us to gain insights into our data set and further has enabled us to recognize the patterns and relationships such as the co-occurrence of adverse birth outcomes among same infants and these infants had mothers with average to a high level of NOx exposure during pregnancy. This is beneficial to further model building and using appropriate models to analyze our data. The insight from the use of interactive PCP leads us to data-driven modelling, to better define future models that consider dependence between birth outcomes.

### Ethics approval and consent to participate

Written, informed consent was obtained from the mother for the children and provided their own consent. Follow-ups of the study were approved by the respective ethics committees at the University of KwaZulu-Natal medical school.

## Supplementary information

SAS JMP Scripts for producing PCP.

## Data Availability

The data that support the findings of this study are available from MACE study. Restrictions apply to the availability of these data, but requests may be made through study management.
