# Supplementary material for: Exploratory Data Analysis of Adverse Birth Outcomes and Exposure to Oxides of Nitrogen Using Interactive Parallel Coordinates Plot Technique
Source: Sci Rep. 2020 Apr 30;10:7363. doi: 10.1038/s41598-020-64471-w (PMC7193633; doi:10.1038/s41598-020-64471-w)
Supplement: Supplementary file 1 — SAS JMP Scripts for producing PCP. [file 41598_2020_64471_MOESM1_ESM.pdf]

Exploratory Data Analysis of Adverse Birth Outcomes and Exposure to Oxides of Nitrogen Using Interactive Parallel Coordinates Plot Technique

*Aweke A. Mitku [1,3], Temesgen Zewotir[1], Delia North [1], Rajen N. Naidoo[2]

[1]School of Mathematics, Statistics and Computer Science, University of KwaZulu-Natal, Durban, South Africa

[2]Discipline of Occupational and Environmental Health, School of Nursing and Public Health, College of Health Sciences, University of KwaZulu-Natal, Durban, South Africa

[3]Department of Statistics, Bahir Dar University, Bahir Dar, Ethiopia.

* Corresponding author

SAS JMP Scripts for producing PCP

```
Graph Builder(
	Size( 1333, 620 ),
	Show Control Panel( 0 ),
	Variables(
		X( :GestationalAge ),
		X( :ChildBirthWeight, Position( 1 ) ),
		X( :ChildBirthLength, Position( 1 ) ),
		X( :HeadCircumference, Position( 1 ) ),
		X( :APGARScore1minute, Position( 1 ) ),
		X( :APGARScore5minute, Position( 1 ) ),
		X( :NOx_Pred__T_, Position( 1 ) )
	),
	Elements(
		Parallel(
			X( 7 ),
```

```
				X( 1 ),
				X( 2 ),
				X( 3 ),
				X( 4 ),
				X( 5 ),
				X( 6 ),
				Legend( 7 )
			)
		),
		SendToReport(
			Dispatch(
				{},
				"GestationalAge",
				ScaleBox,
				{Label Row(
					{Tick Mark(
						Label( "APGARScore1minute" ),
						Label( "APGAR Score 1 minute" )
					), Tick Mark(
						Label( "APGARScore5minute" ),
						Label( "APGAR Score 5 minute" )
					), Tick Mark(
						Label( "ChildBirthLength" ),
						Label( "Child Birth Length" )
					), Tick Mark(
						Label( "ChildBirthWeight" ),
						Label( "Child Birth Weight" )
					), Tick Mark(
						Label( "GestationalAge" ),
						Label( "Gestational Age" )
					), Tick Mark(
						Label( "HeadCircumference" ),
```

```
				Label( "Head Circumference" )
			), Tick Mark( Label( "NOx_Pred__T_" ), Label( "NOx Predicted" ) )}
		)}
	),
	Dispatch(
		{},
		"400",
		ScaleBox,
		{Legend Model(
			7,
			Properties(
				0,
				{Line Color( 13 ), Line Width( 3 ), Transparency( 0.7 )}
			)
		)}
	),
	Dispatch(
		{},
		"graph title",
		TextEditBox,
		{Set Text( "Parallel coordinate plots of NOx with birth outcomes" )}
	),
	Dispatch (
		{},
		"X title",
		TextEditBox,
		{Font Color( 0 ), Set Text( "Birth outcomes" )}
	)
)
);
```
